# Gelsolin-Cu/ZnSOD interaction alters intracellular reactive oxygen species levels to promote cancer cell invasion

**DOI:** 10.18632/oncotarget.10451

**Published:** 2016-07-06

**Authors:** Lalchhandami Tochhawng, Shuo Deng, Ganesan Pugalenthi, Alan Prem Kumar, Kiat Hon Lim, Tuan Zea Tan, Henry Yang, Shing Chuan Hooi, Yaw Chong Goh, Sutherland K. Maciver, Shazib Pervaiz, Celestial T. Yap

**Affiliations:** ^1^ Department of Physiology, Yong Loo Lin School of Medicine, National University of Singapore, Singapore; ^2^ Department of Pharmacology, Yong Loo Lin School of Medicine, National University of Singapore, Singapore; ^3^ Bioinformatics Group, Bioscience Core Laboratory, King Abdullah University of Science and Technology (KAUST), Kingdom of Saudi Arabia; ^4^ Cancer Science Institute of Singapore, National University of Singapore, Singapore; ^5^ National University Cancer Institute, Singapore; ^6^ Curtin Health Innovation Research Institute, Biosciences Research Precinct, School of Biomedical Sciences, Faculty of Health Sciences, Curtin University, WA, Australia; ^7^ Department of Biological Sciences, University of North Texas, Denton, TX, USA; ^8^ Department of Pathology, Singapore General Hospital, Singapore; ^9^ Department of General Surgery, Singapore General Hospital, Singapore; ^10^ Department of General Surgery, Mount Elizabeth Hospital, Singapore; ^11^ Centre for Integrative Physiology, University of Edinburgh, United Kingdom; ^12^ School of Biomedical Sciences, Faculty of Health Sciences, Curtin University, Perth, Western Australia; ^13^ NUS Graduate School for Integrative Sciences and Engineering, National University of Singapore, Singapore; ^14^ Singapore-MIT Alliance, Singapore

**Keywords:** invasion, gelsolin, cytoskeleton, Cu/ZnSOD, ROS, antioxidant

## Abstract

The actin-binding protein, gelsolin, is a well known regulator of cancer cell invasion. However, the mechanisms by which gelsolin promotes invasion are not well established. As reactive oxygen species (ROS) have been shown to promote cancer cell invasion, we investigated on the hypothesis that gelsolin-induced changes in ROS levels may mediate the invasive capacity of colon cancer cells.

Herein, we show that increased gelsolin enhances the invasive capacity of colon cancer cells, and this is mediated via gelsolin's effects in elevating intracellular superoxide (O_2_^.-^) levels. We also provide evidence for a novel physical interaction between gelsolin and Cu/ZnSOD, that inhibits the enzymatic activity of Cu/ZnSOD, thereby resulting in a sustained elevation of intracellular O_2_^.-^. Using microarray data of human colorectal cancer tissues from Gene Omnibus, we found that gelsolin gene expression positively correlates with urokinase plasminogen activator (uPA), an important matrix-degrading protease invovled in cancer invasion. Consistent with the *in vivo* evidence, we show that increased levels of O_2_^.-^ induced by gelsolin overexpression triggers the secretion of uPA. We further observed reduction in invasion and intracellular O_2_^.-^ levels in colon cancer cells, as a consequence of gelsolin knockdown using two different siRNAs. In these cells, concurrent repression of Cu/ZnSOD restored intracellular O_2_^.-^ levels and rescued invasive capacity.

Our study therefore identified gelsolin as a novel regulator of intracellular O_2_^.-^ in cancer cells via interacting with Cu/ZnSOD and inhibiting its enzymatic activity. Taken together, these findings provide insight into a novel function of gelsolin in promoting tumor invasion by directly impacting the cellular redox milieu.

## INTRODUCTION

Metastasis accounts for 90% of solid tumor deaths worldwide [1]. Amongst other factors involved in metastasis, invasion of tissues by cancer cells is a major determinant and the initial step of metastasis. Through attachment to and subsequent degradation of the extracellular matrix (ECM), loss of intercellular adhesion, enhanced cell motility and resistance to death, cancer cells acquire invasive capacity thereby resulting in metastatic spread [2,3]. Central to cancer cell migration and invasion is the regulation of the actin cytoskeleton. The gelsolin superfamily of proteins, in particular gelsolin, is an important regulator of the actin cytoskeleton [4]. Owing to its regulation of actin filaments via capping and severing, gelsolin is believed to promote cell migration and invasion. Gelsolin has been shown to translocate to the plasma membrane and associate with the actin cytoskeleton. Due to this association, the ratio between actin filament and monomeric actin is significantly reduced, which promotes cell migration [5]. The critical involvement of gelsolin in cells' invasive capacity is further corroborated by the findings that cell invasion associated with supervillin, another member of the gelsolin superfamily, also involves the intermediacy of gelsolin [6]. Multiple cell survival/proliferation pathways have been implicated in gelsolin-induced migration and invasion, namely Ras-PI3K-Rac network [7], epidermal growth factor (EGF) [8] and erbB-2/EGFR (epidermal growth factor receptor) [9] pathways.

Cellular protrusions such as lamellipodia, filopodia and invadopodia are critical invasive structures that form the leading edge of invading cells in cancer [10]. Gelsolin has been shown to play an important role in the EGF-induced cell migration by participating in lamellipodia formation [8]. Gelsolin has also been shown to be important for the formation of podosomes in osteoclasts [11], the equivalent structure of invadpodia in non-cancer cells. The role of gelsolin in cellular protrusions can therefore be attributed to its regulation of the actin cytoskeleton. Invasive front in cancer cells are enriched with proteases such as the matrix metalloproteinases (MMPs) and the urokinase plaminogen activator (uPA) that aid in the degradation of the ECM [12,13]. We have previously reported that gelsolin overexpression enhances the production and secretion of uPA, thereby promoting invasion in colon cancer cells [14]. Our finding makes it tempting to speculate a critical role of gelsolin in proteolysis at the invasive front of cancer cells. In addition, it also points to an aspect of gelsolin which may be distinct from its actin regulation. The molecular mechanism by which gelsolin contributes to protease secretion remains poorly understood.

Reactive oxygen species (ROS) are chemically active molecules formed by the incomplete reduction of oxygen (O_2_). Superoxide (O_2_^.-^), the precursor ROS is generated through enzymatic action of NADPH oxidase (Nox) or as a metabolic by-product of mitochondrial respiration. Subsequent reactions can give rise to the formation of other types of ROS such as hydrogen peroxide (H_2_O_2_), hydroxyl radical (^.^OH) and hypochlorous acid (HOCl). Under normal physiological conditions, redox homeostasis is maintained by the efficient cellular antioxidant systems [15]. Whereas, overwhelming oxidative stress is invariably associated with cell and tissue damage and/or death, there is accumulating evidence to implicate mild oxidative stress with cellular proliferation, growth and survival. In this regard, an imbalance between ROS production and anti-oxidant defense mechanisms has been shown to promote cell transformation and oncogenesis [16,17]. In fact, the myriad of signaling networks engaged by a pro-oxidant cellular milieu also provides the cells with the ability to migrate and invade, thereby favoring the progression of the disease [18]. Of note, there is recent evidence to implicate ROS-mediated regulation of the actin cytoskeleton dynamics in enhanced migration and invasion [19,20]. For example, intracellular O_2_^.-^ is implicated in the small GTPase Rac1 mediated increase in actin polymerization. Pharmacological inhibition of intracellular ROS production with the Nox inhibitor diphenyleneiodonium (DPI), as well as the use of ROS scavengers such as manganese (III) tetrakis (1-methyl-4-pyridyl) porphyrin (MnTMPyP), abrogates actin filament elongation [21], thus suggesting the involvement of ROS in actin cytoskeletal dynamics. Furthermore, studies have also indicated that the actin cytoskeleton may initiate ROS generation, actin assembly was shown to enhance O_2_^.-^ production in eosinophils [22]. Stabilization of the Nox complex as well as recruitment of Nox subunits are also known to be influenced by the actin cytoskeletal proteins [23-25]. These findings provide testimony that, in addition to providing structural support, actin and its associated proteins may be engaged in ROS generation that promotes cancer cell invasion. Stimulated by these findings, here we hypothesized that gelsolin, a key actin regulatory protein, may contribute to the control of ROS levels in cancer cells. In this study, we provide evidence that the expression of gelsolin promotes invasion via mechanisms that involve an increase in intracellular O_2_^.-^ in HCT116 colon cancer cells. Gelsolin suppresses the enzymatic activity of Cu/ZnSOD, thereby resulting in an accumulation of O_2_^.-^ in the cells. Bioinformatic analysis and *in situ* approaches reveal the existence of a protein-protein interaction between gelsolin and Cu/ZnSOD that might account for the inhibition of the enzymatic activity. Thus, our findings provide a novel mechanism by which gelsolin mediates colon cancer cell invasion via modulating intracellular O_2_^.-^ levels.

## RESULTS

### Intracellular O_2_^.-^ levels are modulated by gelsolin expression in cells

**Figure 1:**
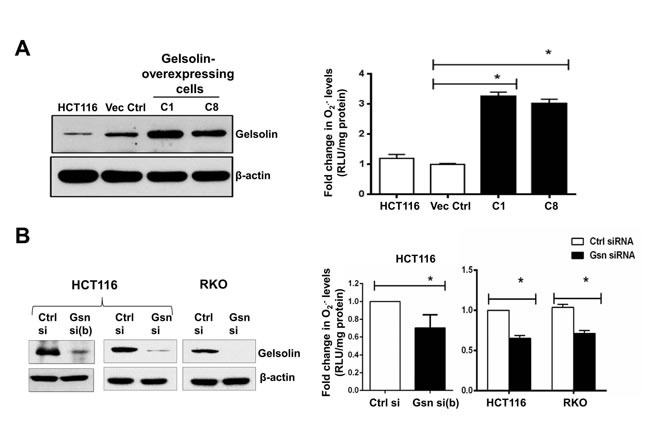
Gelsolin modulates intracellular superoxide (O^.-^) **levels**. (**A**) Left panel: Western blot showing overexpression of gelsolin in HCT116 cells. Right panel: Intracellular O_2_^.-^ levels were measured using the chemiluminescent-based lucigenin assay. Gelsolin-overexpressing cells (C1&C8) show significantly higher levels of O_2_^.-^ when compared to the empty vector control cells. (**B**) Left panel: Western blot showing gelsolin-knockdown in HCT116 and RKO cells using two different siRNAs (Gsn si (b) & Gsn si) targeting gelsolin in HCT116 and a single siRNA (Gsn si) in RKO cells. Right panel: Silencing of gelsolin in HCT116 and RKO cells results in decreased levels of O_2_^.-^ when compared to the control siRNA. O_2_^.-^ data shown are mean ± SD of at least three independent experiments. *p-value <0.05 versus controls using a two tailed Student's *t*-test. Values (mean ± SD ) are expressed as fold over the empty vector control or the control siRNA, which is arbitrarily set to one. The western blot gel pictures are representative images from three independent experiments.

We first sought to determine if gelsolin affects intracellular levels of ROS such as O_2_^.-^ , H_2_O_2_, ^.^OH and HOCl. Using the chemiluminescent based lucigenin assay and the cell permeable dihydroethidium (DHE) dye, we assessed the changes in intracellular O_2_^.-^ levels with increased expression of gelsolin. Under normal growth conditions, the level of O_2_^.-^ was significantly elevated in cells stably overexpressing gelsolin (C1 and C8 cells) when compared to control cells stably transfected with the empty vector (Figures 1A & S1A). Furthermore, siRNA mediated gene silencing of gelsolin in HCT116, RKO, HeLa and HepG2 cells resulted in a significant decrease in intracellular O_2_^.-^ levels (Figures 1B, S1B & C). Taken together, these data provide evidence to link gelsolin expression to an increase in intracellular O_2_^.-^ levels.

Previous studies have shown that several ROS including H_2_O_2_, ^.^OH and HOCl can oxidize 5-(and-6)-chloromethyl-2′,7′-dichlorodihydrofluorescein diacetate acetyl ester (CM-H_2_DCFDA) [26-29]. Hence, gelsolin was depleted in several cancer cell lines including HCT116, RKO, Caco-2, DLD-1, HeLa and HepG2 and CM-H_2_DCFDA oxidation assay was performed. However, there was no significant change in CM-H_2_DCFDA intensity between gelsolin-knockdown and the control siRNA-treated cells (Figure S2A). This indicates that gelsolin is minimally involved in modulating H_2_O_2_, ^.^OH and HOCl levels in these cancer cells. In addition, the Amplex Red H_2_O_2_ assay was used to detect H_2_O_2_ levels in gelsolin-overexpressing cells. The Amplex Red reagent reacts with H_2_O_2_ resulting in a red fluorescent oxidation product which could be assayed fluorometrically. Again, no significant difference was observed in H_2_O_2_ levels when gelsolin expression was increased, compared to the empty vector transfected cells (Figure S2B). Taken together, these data suggest that gelsolin overexpression may specifically modulate only the levels of intracellular O_2_^.-^.

### Gelsolin modulates O_2_^.-^ levels by suppressing Cu/ZnSOD activity

**Figure 2:**
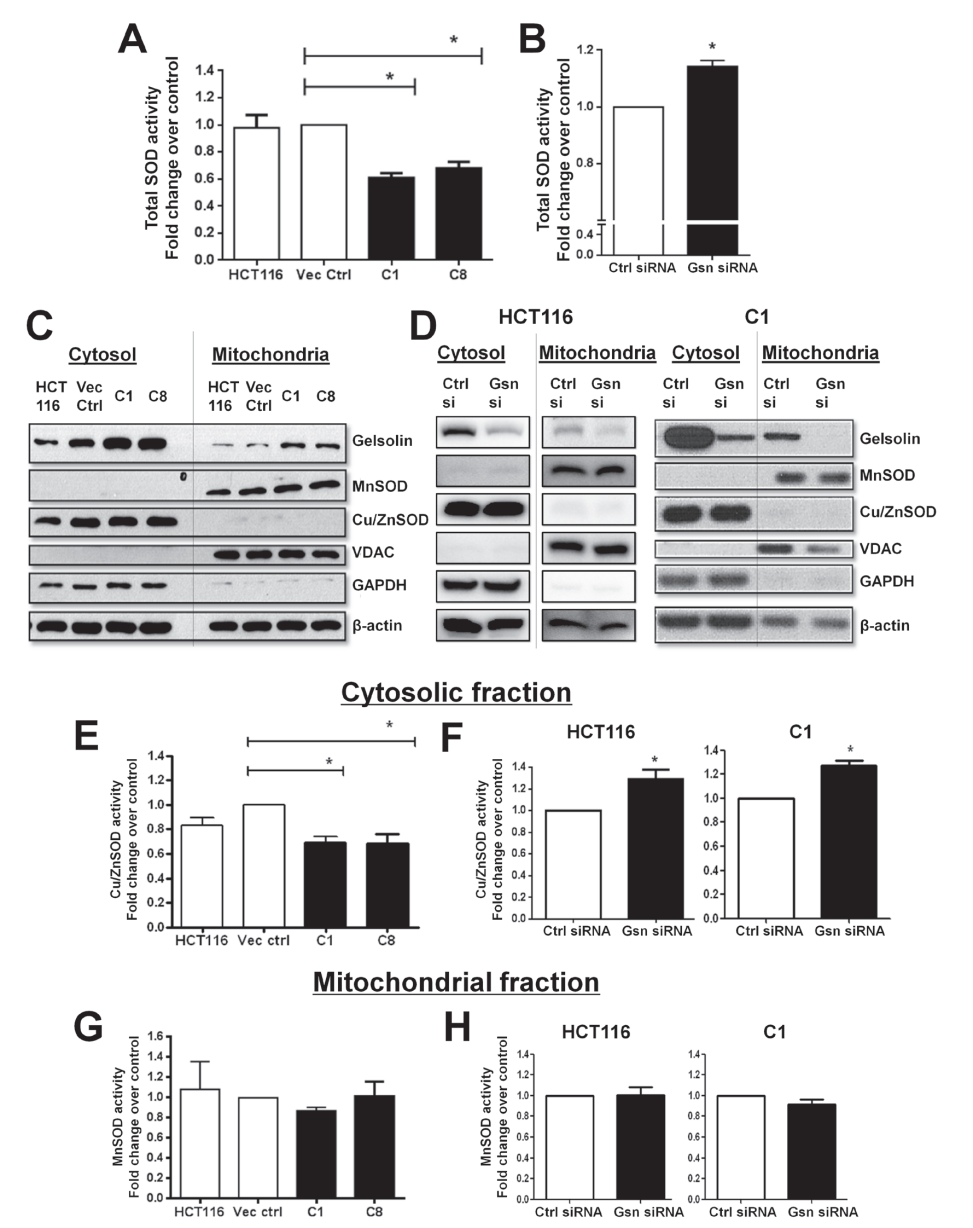
Gelsolin modulates SOD activity (**A** & **B**) Total intracellular SOD activity was measured from total cell lysates. Increased gelsolin expression lowers the SOD activity in both C1 and C8 cells as compared to wild type HCT116 and empty vector control cells. (B) Knockdown of endogenous gelsolin in HCT116 cells shows increased SOD activity when compared to the control siRNA treated cells. (**C** & **D**) Cytosolic and mitochondrial fractions were isolated in HCT116 cells following either gelsolin overexpression or gelsolin knockdown. Fractionation was confirmed by the presence of MnSOD and VDAC in the mitochondria and Cu/ZnSOD and GAPDH in the cytosol. β-actin was used as the internal loading control. (**E** & **F**) Cu/ZnSOD activity was measured from the cytosolic extract. Gelsolin overexpression results in suppression of Cu/ZnSOD activity when compared to the empty vector control cells whereas knockdown of gelsolin in HCT116 and C1 cells results in elevated Cu/ZnSOD activity. (**G** & **H** ) MnSOD activity was determined from the mitochondrial fraction. No significant change in MnSOD activity was observed when gelsolin was overexpressed or silenced in HCT116 and C1 cells. *p-value <0.05 versus controls using a two tailed Student's t-test. Values (mean ± SD ) are expressed as fold over the empty vector control or the control siRNA, which is arbitrarily set as one. The western blot gel pictures are representative images from three independent experiments.

Antioxidants play important roles in maintaining the cellular redox homeostasis, therefore, abnormal fluctuations in the antioxidant system significantly alters the ROS-antioxidant balance resulting in redox dyshomeostasis [30]. An important determinant of increased O_2_^.-^ levels in cells is the loss of enzymatic activity of the antoxidant Cu/ZnSOD [31]. As such, we questioned whether the increase in intracellular O_2_^.-^ upon overexpression of gelsolin was a function of a compromise in the enzymatic activity of Cu/ZnSOD. To assess that, SOD activity was first measured in total cell lysate (this is the combined enzymatic activities of the two SOD isoforms , Cu/ZnSOD and MnSOD). Indeed, total SOD activity was significantly lower in the two cell lines overexpressing gelsolin, (C1 and C8), compared to the empty vector transfected and wild-type HCT116 cells (Figure 2A). Similarly, depletion of endogenous gelsolin expression in wild-type HCT116 results in higher total SOD activity, relative to the control siRNA transfected cells (Figure 2B).

In order to identify the SOD isoform targeted by gelsolin, we performed subcellular fractionation experiment that separates the cytosol from intact mitochondria. Cu/ZnSOD and MnSOD activities were assayed from the respective fractions. The validity of our fractionation was confirmed by the presence of Cu/ZnSOD and GAPDH in the cytosol and MnSOD and VDAC in the mitochondrial fractions, whilst β-actin was used as the internal loading control for both fractions (Figure 2C & 2D). Gelsolin-overexpressing C1 and C8 cells displayed lower Cu/ZnSOD activity compared to empty vector control and wild-type HCT116 cells (Figure 2E), whereas Cu/ZnSOD activity was significantly elevated following gelsolin knockdown in both HCT116 and C1 cells (Figure 2F). However, MnSOD activity was only minimally affected by the modulation of gelsolin expression as shown in Figure 2G & 2H. These results indicate that changes in gelsolin expression alter Cu/ZnSOD activity but not MnSOD activity, and therefore the reduction of total SOD activity observed in Figure 2A is mainly accounted by the loss of Cu/ZnSOD activity. It should be pointed out that changes in enzymatic activites could be influenced by protein expression; however the protein levels of both Cu/ZnSOD and MnSOD were not affected upon modulation of gelsolin expression (Figure 2C & 2D).

### *In silico* analysis of gelsolin and Cu/ZnSOD interaction

**Figure 3:**
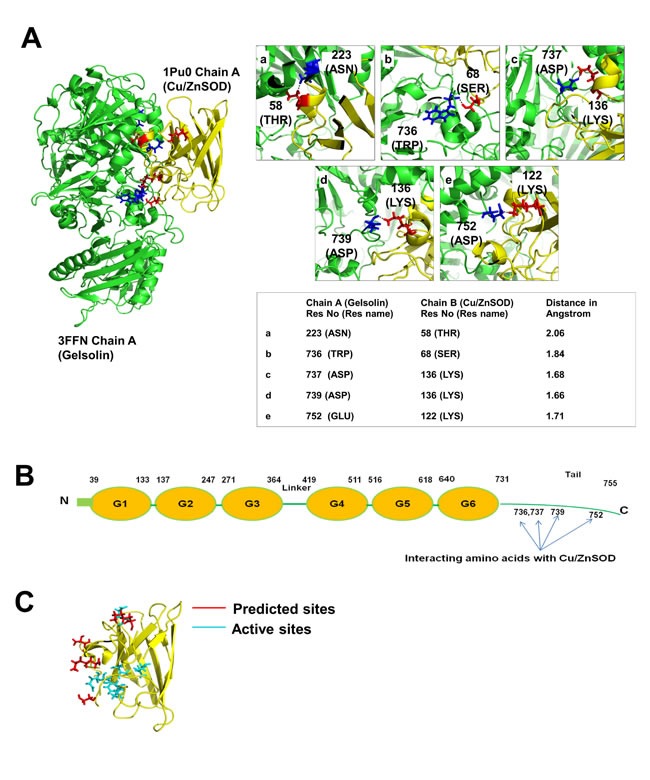
*In silico* analysis of gelsolin and Cu/ZnSOD interaction (**A**) 3-dimensional structure of gelsolin-Cu/ZnSOD complex was obtained using PatchDock analysis. The green structure corresponds to gelsolin (PDB: 3FFN, chain A) and the yellow structure represents the Cu/ZnSOD structure (PDB: 1PU0 Chain A). The interacting region of the complex structure of Gelsolin-Cu/ZnSOD was analyzed using PyMol software. The interacting residues were identified and the distance between these residues were calculated and represented in angstrom. (**B**) Cartoon model of gelsolin structure with domains and the C-terminal tail region. The C-terminal tail region of gelsolin is important for its interaction with Cu/ZnSOD. Four of the amino acids at positions 736, 737, 739 and 752 of the C-terminal tail region of gelsolin participate in the complex formation with Cu/ZnSOD. (**C**) The predicted amino acid residues (positions 58, 68, 136 and 122) of Cu/ZnSOD that participate in its interaction with gelsolin lie close to the active sites of the Cu/ZnSOD (47,49,64,81,84,121) in the 3-Dimesional structure.

In our attempt to explore how gelsolin suppresses Cu/ZnSOD activity, we tested the possibility of a protein-protein interaction between gelsolin and Cu/ZnSOD. Docking analysis using PatchDock was performed between gelsolin (PDB: 3FFN, chain A) [32] and Cu/ZnSOD (PDB: 1PU0 Chain A) [33], which suggested the presence of a direct interaction between gelsolin and Cu/ZnSOD (Figure S3A). In addition, we identified the amino acid residues involved in the interaction (Figure 3A-C), which also provided evidence that the C-terminus of gelsolin is important in its interaction with Cu/ZnSOD (Figure 3B). The amino acid residues 736, 737, 739 and 752 of gelsolin were predicted to form polar bonds with the amino acid residues 68, 136, 136 and 122 of Cu/ZnSOD, respectively. Using Pymol, a molecular visualization tool, the distances between the predicted interacting amino acid residues were found to be less than 2 angstroms (Figure 3A), suggesting that these amino acid residues are in close spatial proximity, and thus polar bonds can possibly form between these two proteins. Moreover, the predicted amino acid residues within Cu/ZnSOD that participate in the complex formation lie very close to the enzymatic active site of Cu/ZnSOD [34] (at amino acid positions 47,49,64,81,84,121 [http://www.ncbi.nlm.nih.gov/protein/CAG46542.1]) (Figure 3C). It is therefore probable that the complex formation and the 3-dimensional folding of the proteins hinder the catalytic activity of Cu/ZnSOD. Moreover, the stability of the docked gelsolin-Cu/ZnSOD complex was analyzed with molecular dynamics simulation that mimics physiological conditions. Molecular dynamics simulation shows that the gelsolin-Cu/ZnSOD complex structure remains intact for up to 10 nanoseconds (Figure S3B). Taking together, our *in silico* analysis suggests that gelsolin and Cu/ZnSOD are potential binding partners, and they likely form stable complexes under physiological conditions. Considering the predicted binding site on Cu/ZnSOD catalytic site, it is possible that the potential interaction with gelsolin may influence Cn/ZnSOD activity.

### Gelsolin interacts with Cu/ZnSOD *in situ* and *in vitro*

**Figure 4:**
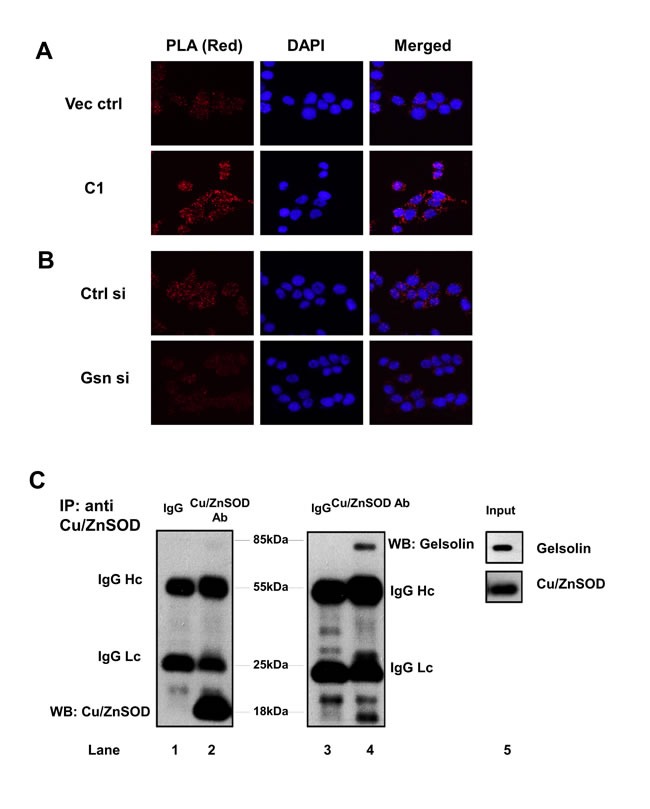
Gelsolin forms a complex with Cu/ZnSOD (**A**-**B**) Proximity ligation assay (PLA) was performed in gelsolin-overexpressing and gelsolin-knockdown cells. PLA signals in red fluorescence were detected when cells were treated with both gelsolin and Cu/ZnSOD antibodies. Nuclei were counterstained with DAPI. Images were captured using Olympus DP72 microscope and cellSens software at 40X. (**C**) Endogenous Cu/ZnSOD was immunoprecipitated from lysates of C1 cells using Cu/ZnSOD antibody. Cu/ZnSOD and gelsolin were detected in the western blot using specific antibodies against Cu/ZnSOD and gelsolin as shown in lanes 2 and 4. The negative mouse IgG control in lanes 1 and 3 does not detect any protein band. Lane 5 shows the protein expression of gelsolin and Cu/ZnSOD in the C1 cell lysate. Data shown here is a representative of three independent experiments.

To validate our *in silico* analysis, we performed an *in situ* proximity ligation assay (PLA) as well as coimmunoprecipitation (Co-IP) studies between gelsolin and Cu/ZnSOD. Permeabilized cells were treated with primary antibodies against gelsolin and Cu/ZnSOD followed by treatment with secondary antibody probes. The probes are attached to short DNA strands or oligos. If the two proteins interact with each other, the oligos from both the probes ligate, and upon amplification emit a fluorescent signal. The close association between gelsolin and Cu/ZnSOD was confirmed upon detection of the PLA signal in red fluorescence, which was visualized using fluorescent microscopy at 563nm (Figure 4A). The intensity of the red signal observed in C1 cells (overexpressing gelsolin) are higher compared to the vector control cells, thus indicating a greater association between gelsolin and Cu/ZnSOD. Negative controls such as treatment with isotype antibody and single specific primary antibody (ie. either anti-gelsolin or anti-Cu/ZnSOD antibody alone) were included to confirm the specificity of the assay. No red fluorescent signals were detected in the negative control sets (Figure S4). Importantly, when we depleted gelsolin expression in C1 cells, the intensity of the red signal became significantly weaker in gelsolin-knockdown cells, relative to the control siRNA-treated cells (Figure 4B). To further confirm if gelsolin interacts with Cu/ZnSOD, endogenous Cu/ZnSOD was immunoprecipitated from the lysate of C1 cells. The presence of gelsolin in the immunoprecipitate was detected by western blot (Figure 4C), thus confirming that there is physical interaction between the two proteins.

### Gelsolin mediates colon cancer cell invasion via modulating the intracellular O_2_^.-^ levels

**Figure 5:**
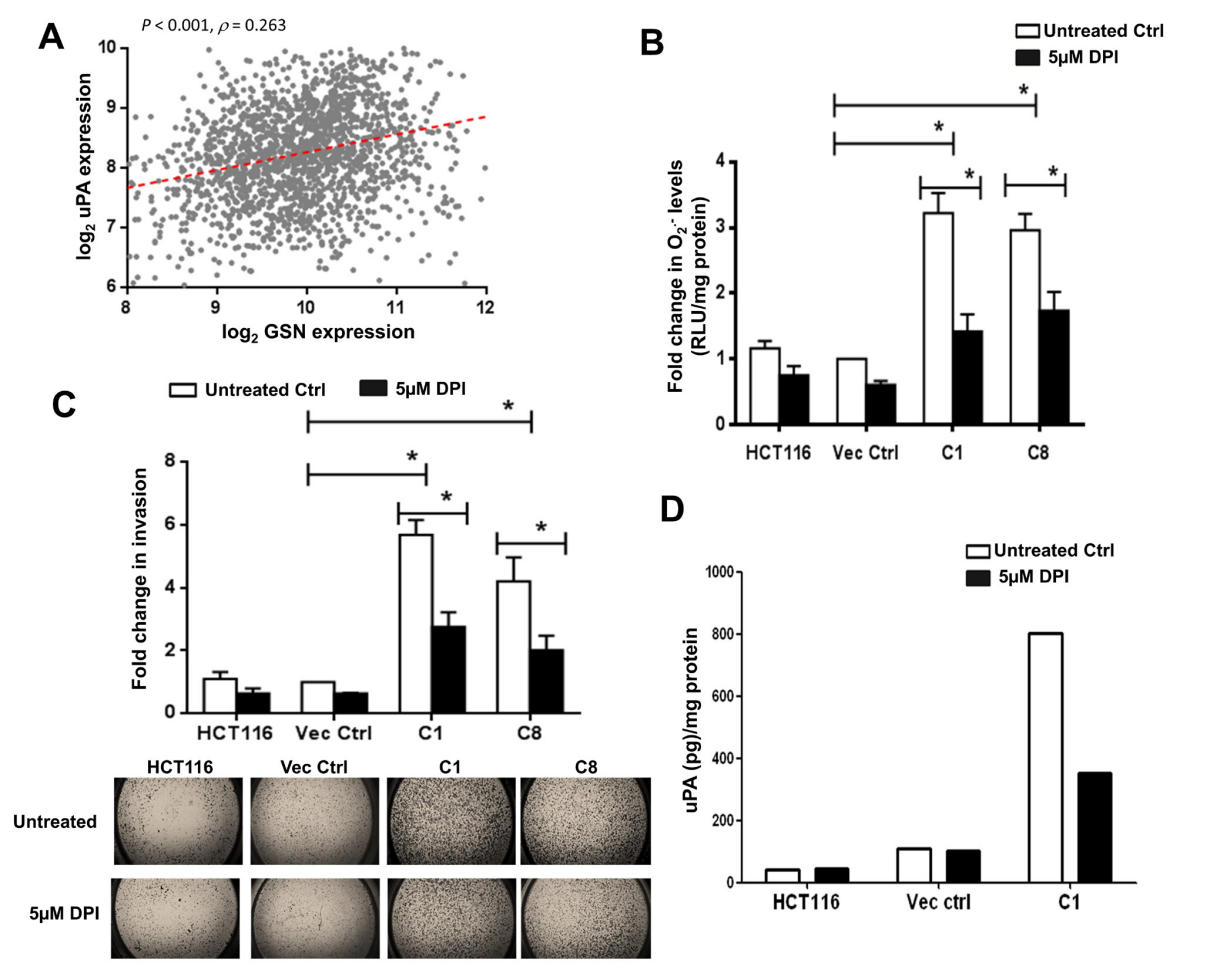
DPI inhibits gelsolin-induced O_2_^.-^ invasion and uPA levels (**A**) Correlation of gelsolin expression with uPA across 1820 colorectal cancer samples. Spearman's rank correlation test was used to access the correlation of uPA expression with gelsolin. The correlation coefficient (*ρ*) and its significance (P value) are indicated. (B-D) Treatment of cells with 5μM DPI for 24 hours significantly lowered (**B**) O_2_^.-^ levels and (**C**) invasion of gelsolin-overexpresing cells and (**D**) gelsolin-induced uPA secretion. Upper panel B, quantitative representation of invaded cells following 5μM DPI treatment. Lower panel B, representative pictures of invaded cells with or without DPI treatments are shown (2.5X magnification of the entire well). *p-value <0.05 versus controls using a two tailed Student's t-test. Values (mean ± SD ) are expressed as fold over the empty vector control, which was arbitrarily set as one. (**D**) Cells were serum starved with or without 5μM DPI for 8 hours and the conditioned media were used to detect uPA by ELISA. Treatment of cells with 5μM DPI significantly inhibited uPA secretion in the gelsolin-overexpressing C1 cells whereas 5μM DPI treatment minimally affected uPA secretion in the empty vector control and wild-type HCT116. Secreted uPA levels were normalized to protein concentration. The data shown here is the raw ELISA reading and a representative of three independent experiments.

We have previously shown that increased gelsolin expression promotes colon cancer cell invasion through its involvement in the secretion and increased activiy of uPA [14]. To understand the involvement of gelsolin in uPA-mediated invasion in colorectal cancer *in vivo*, we analyzed the correlation between gelsolin and uPA gene expression. Based on the microarray data of tumor tissues from Gene Omnibus (GEO), gene expression of gelsolin showed a signifcant positive correlation with uPA in colorectal cancer (Figure 5A). Furthermore, gelsolin expression was found to correlate with several genes involved in epithelial–mesenchymal transition (EMT) (Figure S5), a process promoting invasion [35]. These data strongly support the pro-invasive role of gelsolin in colorectal cancer.

To gain a deeper understanding of the role of geloslin in invasion, we tested if its regulation of O_2_^.-^ contributes to gelsolin-mediated invasion. We used 5μM diphenyleneiodonium(DPI), a potent inhibitor of Nox [36] to inhibit O_2_^.-^ production in the cells. Treatment of cells with 5μM DPI significantly abrogated O_2_^.-^ production in gelsolin-overexpressing C1 and C8 cells (Figure 5B). The role of O_2_^.-^ in gelsolin-mediated invasion was further assessed using a transwell invasion assay, performed under similar conditions of DPI treatment. Consistent with the reduction in O_2_^.-^ levels, the invasive capacity of gelsolin-overexpressing cells was significantly decreased following DPI treatment (Figure 5C). It should be pointed out that the reduction in invasion observed upon DPI treatment was not due to reduction in cell viability as assessed using the trypan blue dye exclusion method; 5μM DPI for 24 hours had minimal effect on cell viability (Figure S6). We also tested if O_2_^.-^ participated in enhancing uPA secretion induced by gelsolin. Cells were serum starved with or without 5μM DPI for 8 hours and the conditioned media were used for the detection of uPA by ELISA. Treatment of cells with DPI significantly inhibited uPA secretion in C1 cells, whereas similar treatment had a minimal effect on the empty vector transfected and wild-type HCT116 cells (Figure 5D). We also confirmed that the cell viability was not affected by the DPI treatment (Figure S7). Taken together, these data implicate the role of intracellular O_2_^.-^ in gelsolin-induced tumor cell invasion and uPA secretion.

**Figure 6:**
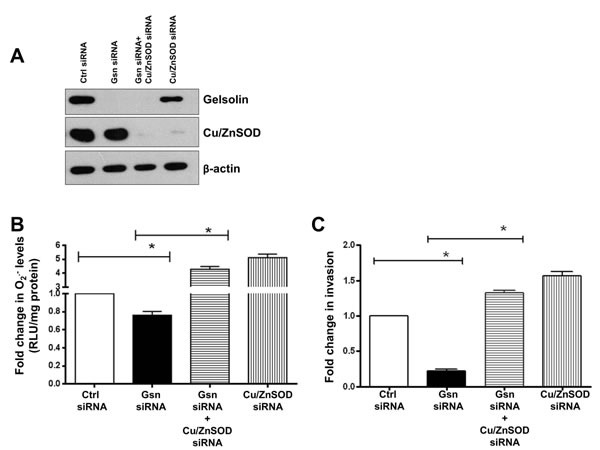
O_2_^.-^ rescues invasion in gelsolin-depleted cells O_2_^.-^ levels were induced in gelsolin-knockdown cells by silencing Cu/ZnSOD in wild-type HCT116 cells. (**C**) Western blot showing knockdown of gelsolin and Cu/ZnSOD in HCT116 cells. (**B**) Simultaneous knockdown of gelsolin and Cu/ZnSOD significantly increases O_2_^.-^ levels in gelsolin-depleted cells. (**C**) Increasing O_2_^.-^ levels by knockdown of Cu/ZnSOD rescues invasion in gelsolin-depleted HCT116 cells. Data shown are mean ± SD of at least three independent experiments. *p-value <0.05 versus controls using a two tailed Student's ***t***-test. Values (mean ± SD ) are expressed as fold over the empty vector control or the control siRNA, which is arbitrarily set to one. The western blot gel picture is a representative image from three independent experiments.

To confirm the requirement of O_2_^.-^ in tumor cell invasion, we asked if an increase in O_2_^.-^ could phenocopy the effect of gelsolin in cells where the expression of gelsolin had been repressed by siRNA-mediated gene silencing. Therefore, the expression of Cu/ZnSOD was silenced by siRNA in gelsolin-depleted cells. Silencing of Cu/ZnSOD abrogated the conversion of O_2_^.-^ to H_2_O_2_ thus leading to increased accumulation of O_2_^.-^ even in the absence of gelsolin. Knockdown of gelsolin alone resulted in a decrease in intracellular O_2_^.-^, however, simultaneous knockdown of gelsolin and Cu/ZnSOD restored O_2_^.-^ levels in gelsolin-depleted cells (Figure 6A & 6B). Similarly, knockdown of gelsolin results in decreased invasion, however, simultaneous knockdown of Cu/ZnSOD and gelsolin was able to restore invasion in gelsolin-depleted cells (Figure 6C). These data provide strong evidence to link intracellular O_2_^.-^ to the invasive capacity of tumor cells as well as underpin the requirement of O_2_^.-^ in gelsolin-induced cancer cell invasion.

## DISCUSSION

The role of gelsolin in cancer has been controversial due to conflicting evidence suggesting gelsolin's role as a tumor suppressor or activator. The tumor suppressive role of gelsolin is mostly linked to its downregulation in several cancers including breast [37], colon [38], stomach [39], bladder [40], prostate [41], lung [42] as well as in transformed human fibroblast and epithelial cells [43]. Conversely, a number of studies have shown the tumorigenic potential of gelsolin [44-47], including our report on gelsolin's role in inducing uPA secretion to promote tumor cell invasion [14]. In this study, we explored the mechanisms by which gelsolin exert its oncogenic behaviour, specifically in promoting the invasive phenotype. We provide evidence that increased gelsolin expression in HCT116 cells creates a pro-oxidant milieu and promotes tumor cell invasion.

Oxidative stress has been shown to be associated with aggressive phenotypes in cancer cells [48, 49] and cancer cell fate decisions are regulated by the cellular redox environment. Whilst a sublethal increase in oxidants may promote cancer cell growth, proliferation and invasion, excessively high oxidative stress is associated with cell and tissue injury and death [50-52]. We show that overexpression of gelsolin generates a sublethal level of oxidative stress that confers an invasive phenotype. Blockade of O_2_^.-^ production with DPI attenuated the invasive capacities of gelsolin-overexpressing cells as well as lowering gelsolin-induced uPA secretion, implying the critical intermediacy of O_2_^.-^ in gelsolin-induced invasion. In addition to cancer cell invasion, the oncogenic property of O_2_^.-^ has been evidenced through earlier studies that linked oncogene-induced cell survival and proliferation to an increase in intracellular O_2_^.-^ [53-56]. Our findings thus corroborate the oncogenic behavior of O_2_^.-^ in that it facilitates gelsolin-induced invasion in HCT116 cells.

ROS have been implicated in the alteration of the tumor microenvironment. ROS are well known transcriptional activators of ECM enzymes, such as uPA and MMP family of proteases [57, 58]. In addition, ROS can also directly activate MMPs by oxidative modification of the cysteine residue [59, 60]. However, the effect of intracellular ROS on the enzymatic activity of uPA is not well understood. We have previously reported that gelsolin upregulated the mRNA and protein levels of uPA as well as induced its enzymatic activity [14]. Moreover, our clinical data from colorectal cancer patient gene expression profiles reveals a positive corelation between gelsolin expression and uPA expression, suggesting a clinical significance of gelsolin in promoting cancer invasion. Results presented in this report not only corroborate our earlier findings, but also provide a mechanistic insight into the effect of gelsolin on uPA expression and activity by demonstrating the requirement of intracellular O_2_^.-^ in gelsolin-induced uPA upregulation. Furthermore, gelsolin-induced uPA secretion and invasion could be dramatically attenuated upon pharmacological inhibition of Nox with DPI. Although, a number of downstream targets of ROS in cancer has been reported, the upstream events that regulate ROS are not well characterized. Our study reveals gelsolin as a novel upstream inducer of O_2_^.-^ in cancer cells as well as provide evidence to link gelsolin-induced invasion to an increase in intracellular O_2_^.-^. Taken together, our study provides in-depth mechanism in gelsolin-mediated invasion through modulating intracellular O_2_^.-^ levels to enhance uPA secretion and invasion, and this pathway may contribute to invasion and dissemination of cancer in colorectal cancer patient.

By keeping a tight check on the cellular oxidant levels, antioxidants protect cells against oxidant mediated insults. Interestingly, a pro-oxidant intracellular milieu is typically associated with cancer cells, which has been attributed in part to the downregulation of antioxidant defense systems in transformed or malignant cells. For example, downregulation of the three major antioxidants Cu/ZnSOD, MnSOD and catalase have been reported in prostate cancer [61]. On the other hand, overexpression of Cu/ZnSOD, MnSOD and catalase lowered both the growth rate as well as the aggressive nature of breast cancer cells [48, 49]. SODs act by dismutating O_2_^.-^to H_2_O_2_ [62], thereby acting as a first line of defense in cells. Whilst the enzymatic functions of the two isoforms of SOD, the cytosolic Cu/ZnSOD and mitochondrial MnSOD, are similar, their site of action is restricted by their sub-cellular localization. Cu/ZnSOD is responsible for eliminating O_2_^.-^ in the cytosol and MnSOD scavenges O_2_^.-^ in the mitochondria [17]. Our results point to the suppression of cytoplasmic Cu/ZnSOD activity, but not of its mitochondrial counterpart, MnSOD, which accounts for the rise in the intracellualr levels of O_2_^.-^ in gelsolin-overexpressing cells. Cu/ZnSOD activity is controlled by several factors including copper loading via the chaperone protein CCS (Copper chaperone for Cu/ZnSOD), dimerization of the Cu/ZnSOD molecules and structural stability offered by zinc ions [63, 64]. Besides the biochemical influences, Cu/ZnSOD activity is also determined by its transcriptional regulation and protein expression. In this study, we identified gelsolin as a new regulator of Cu/ZnSOD by directly interacting with and inhibiting its enzymatic function. Our results were substantiated by *in silico* analysis, Co-IP as well as PLA data that confirm the existence of a protein-protein interaction between gelsolin and Cu/ZnSOD under basal cell culture conditions. Further analysis of this interaction from our *in silico* data suggested that the predicted amino acid residues of Cu/ZnSOD that participate in the complex lie close to the enzymatic active sites of Cu/ZnSOD. Judging from the docked gelsolin-Cu/ZnSOD complex structure, Cu/ZnSOD is positioned in a way that both the predicted interacting amino acid residues and the active sites of Cu/ZnSOD (residues 47,49,64,81,84 and 121) faced the groove of the relatively bigger gelsolin molecule and perhaps buried itself into this grove. Considering the relative difference in the molecular sizes of gelsolin (85Kda) and Cu/ZnSOD (18Kda) and the orientation of the molecules, it is plausible that this interaction masks the enzymatic activity of Cu/ZnSOD, which in turn results in an increase in O_2_^.-^ levels.

Structurally, gelsolin is made up of six domains (G1-G6) that are arranged as tandem repeats [65]. Attached to domain six is the C-terminal tail that plays a pivotal role in regulation of actin binding capability of gelsolin. The C-terminal segment of gelsolin binds to actin in the presence of calcium, while binding of N-terminal segment is controlled by both calcium and the C-terminal tail. In the absence of calcium, however, the tail binds G2 (the second gelsolin repeat present in N-terminal half of gelsolin) and prevents binding to actin [66, 67]. Calcium is a critical molecule involved in ROS production [68-70], however, we did not observe significant differences in calcium levels between vector control and gelsolin-overexpressing cells (Figure S8), indicating that gelsolin may not regulate calcium levels in our model. From our *in silico* analysis, it is interesting to note that the C-terminal tail region of gelsolin is likely to be crucial in its binding to Cu/ZnSOD. Interestingly, residues 736, 737, 739 and 752 in gelsolin identified from *in silico* analysis as potential Cu/ZnSOD binding sites all lie at the tail region. Considering the importance of the tail region of gelsolin in its actin-binding regulation, it would be interesting to study if the interaction between gelsolin and Cu/ZnSOD could affect the actin-regulation ability of gelsolin. Taken together, *in silico* analysis of the interaction sites between gelsolin and Cu/ZnSOD reveals that the binding may affect the activities of both gelsolin and Cu/ZnSOD, and it might be interesting to study if the interaction could influence the activities of both proteins which in turn affect the invasive capacity of cancer cells.

In conclusion, our study shows a mechanistic insight into the role of gelsolin in cancer cell invasion. Notably, we have delineated a new role of gelsolin in creating a pro-oxidant milieu that favors tumor cell invasion. We further demonstrate a novel protein-protein interaction between gelsolin and the antioxidant Cu/ZnSOD, which appears to inhibit Cu/ZnSOD activity, leading to increased O_2_^.-^ levels. Disruption of the gelsolin-Cu/ZnSOD complex by small molecules would restore Cu/ZnSOD activity thereby reducing intracellular levels of O_2_^.-^ which could have potential implications for the design of therapeutic strategies to combat tumor invasive capacity.

## MATERIALS AND METHODS

### Cell lines and culture conditions

Human colorectal cancer cell lines HCT116, RKO, Caco-2 and DLD-1 were obtained from ATCC (Manassas, VA, USA). HCT116 was cultured in McCoy's 5A modified medium; RKO, HeLa and HepG2 cells were cultured in Dulbecco's Modified Eagle's Medium (DMEM). McCoy's 5A and DMEM were purchased from Sigma-Aldrich, LO, USA. Stable HCT116 cell lines overexpressing gelsolin, namely C1 and C8 as well as empty vector control cells were grown in McCoy's 5A supplemented with 500 μg/mL Geneticin G418 (Gibco, NY, USA). All the media were supplemented with 10% fetal bovine serum (FBS) (Hyclone, UK). Cells were maintained at 37^o^C in a humidified incubator with 5% CO_2_.

### Cell transfection

Stable cell lines overexpressing gelsolin (C1 and C8) were previously derived from HCT116 cells in our laboratory [14].

Two siRNA duplex oligonucleotides AAACGUCCAAUCUUGUUGGAGCAGG and TAGAACTGTCCATATGTGGCAGGGT (Life Technologies, Carlsbad, CA, USA) at 10nM were used to silence the expression of gelsolin, and the oligonucleotides CCATGCAGGTCCTCACTTTA (Qiagen, Hilden, Germany) was used for the silencing of Cu/ZnSOD following the manufacturer's protocol. Non-targeting siRNA control with medium GC content (Life Technologies) was used as a negative control for gelsolin, and an all star negative control siRNA (Qiagen) was used as a negative control for Cu/ZnSOD. To control for double knockdown, cells were treated with both the control siRNAs (medium GC and all star negative).

### Determination of intracellular ROS

Total intracellular superoxide was measured using the chemiluminescence-based lucigenin method as described [71]. Cells were cultured in complete media under normal growth conditions. Pelleted cells were lysed with somatic cell ATP-releasing agent (Sigma-Aldrich, LO, USA). 400μL of the lysate was immediately transferred to a glass tube and chemiluminescence was monitored using a Berthold Sirius Luminometer. Data were described as Relative Light Units/second/milligram of protein (RLU/s/mg protein). Readings were normalized with protein concentration.

DHE and CM-H_2_DCFDA staining were performed to detect O_2_^.-^ and other ROS respectively. Cells were loaded with either DHE/CM-H_2_DCFDA (Life Technologies) at a final concentration of 10μM at 37ºC for 10 min and analysis was carried out with flow cytometry (BD Facs Calibur/Cyan ADP Beckman Coulter) with excitation/emission at 488/518nm for CM-H_2_DCFDA and 530/610nm for DHE. Data were analyzed using the Cell Quest Pro and Summit softwares.

Specific H_2_O_2_ levels in gelsolin-overexpressing cells were analysed using the Amplex Red Hydrogen Peroxide/Peroxidase Assay Kit (Life Technologies). Cells were lysed and 20μL of cell lysate was dispensed in a well of 96-well plate and Amplex Red reagent mix was added to the samples. Fluorometric reading was immediately determined using Varioskan fluorometric reader at 585nm. Data was normalized to protein concentration.

### Cell viability assay

Cells were cultured with or without 5μM DPI (Sigma-Aldrich) for 24 h (in medium containg 1% FBS) or 8 h (in serum-free medium). Cells were then trypsinized and number of viable cells were counted using trypan blue exclusion method.

### Matrigel invasion assay

Matrigel invasion assay was performed using the BD Matrigel Basement Membrane Matrix (BD Biosciences, CA, USA). 40μL of diluted matrigel (0.33mg/mL, diluted in serum free media) was coated on a 8-μm pore size 6.5-mm diameter transwell filter membrane (Corning, NY, USA). The matrigel was allowed to polymerize at 37^o^C for 2 h. 2 x 10^5^ cells with or without 5μM DPI were seeded on top of the matrigel layer. Complete media containing 10% FBS was added to the lower chamber as a chemoattractant and incubated for 24 h at 37^o^C. Invaded cells were fixed with 70% ethanol for 20 min at room temperature and stained with 0.2% crystal violet for 30 min at room temperature. Invaded cells were then captured using Canon powershot A640 camera at 20X magnification for counting. At least ten representative fields were captured per membrane and the number of invaded cells were manually counted using Metamorph software and quantified.

### Western blotting

Cells were lysed with Radioimmunoprecipitation assay buffer (RIPA) (Sigma-Aldrich) supplemented with protease inhibitors (Roche Complete protease inhibitor cocktail, Roche, Basel, Switzerland). Equal amount of lysates were separated by SDS-PAGE and transferred to a polyvinylidene difluoride (PVDF) membrane (Millipore, MA, USA). Membranes were blocked with 5% w/v milk (Blocking grade milk, Bio-Rad) for 1 h and incubated overnight at 4^o^C with primary antibodies against gelsolin (Abcam, UK), Cu/ZnSOD (Cell Signaling, MA, USA), MnSOD (BD), VDAC (Cell Signaling); GAPDH (BD Biosciences) and β-actin (Sigma-Aldrich). The membranes were washed and incubated with horse radish peroxidase-conjugated secondary antibodies. Signals were visualized using chemiluminescence substrate (Thermo Scientific, MA, USA).

### Immunoprecipitation

Cells were lysed with modified RIPA buffer (5M Nacl, 1M Tris-HCL, pH 8.0, 0.5M EDTA, 1% Triton X-100, 0.1% sodium deoxycholate and protease inhibitors). 500μg of cell lysate was incubated overnight with 20μL of protein A/G agarose beads (Santa Cruz, TX, USA) conjugated to 2μg of primary antibody against Cu/ZnSOD at 4°C. The beads were washed three times with modified RIPA buffer, boiled for 10 min and the proteins were fractionated by SDS-PAGE followed by western blotting.

### *In situ* proximity ligation assay

*In situ* Proximity Ligation Assay (PLA) was performed using the 563 Duolink detection kit (OLINK, Uppsala, Sweden) according to manufacturer's instructions. 1 x 10^5^ cells were grown on a 12-cm coverslip for 24 h. Cells were fixed with 4% parafolrmaldehyde for 20 min followed by blocking using 5% BSA for 1 h at room temperature. Cells were then incubated with primary antibodies against gelsolin and Cu/ZnSOD at 4°C overnight. Cells were washed and incubated with the PLA probes at 37°C for 1 h. The oligonucleotides were then ligated using the ligation reaction mixture at 37°C for 30 min and the ligated product was amplified using the red fluorescent amplification reagent at 37°C for 100 min. Cells were washed and the coverslip was mounted onto a glass slide using mounting media containing DAPI to counterstain the nuclei. Images were captured using Olympus DP72 microscope and cellSens software at 40X and 60X magnification.

### Isolation of intact mitochondria and cytosolic fraction

Intact mitochondria and cytosolic fractions were isolated as described previously [72]. Cells were grown to 70% confluence in a 10-cm tissue culture dish. 150μL of mitochondrial extraction buffer (200mM mannitol, 68mM sucrose, 50mM Pipes-KOH pH 7.4, 50mM KCl, 5mM EGTA, 2mM MgCl2 and 1mM dithiothreitol), containing protease inhibitors was added to the dishes and cells were detached by scraping. The detached cells were transferred to 1.5mL tubes and incubated on ice for 20 min. Cells were homogenized with a dounce homogenizer and passaged for 40 strokes followed by centrifugation at 300 g for 10 min at 4ºC. The supernatant was transferred to a fresh eppendorf tube and centrifuged at 12000g for 30 min at 4ºC. The pellet contains the intact mitochondrial fraction. The supernatant was again centrifuged at 25,000g for 45 min to obtain the cytosolic fraction.

### Superoxide dismutase (SOD) activity assay

SOD activity was measured using the calorimetric-based SOD activity kit (ENZO Life Sciences, NY, USA) according to the manufacturer's protocol. Cells were lysed and 20μg protein from each sample was dispensed to a clear bottom 96-well plate. 150μL of WST-1 and Xanthine oxidase (in a master mix) was pipetted into each well and the reaction was then initiated by adding 25μL of 1X Xanthine Solution. Absorbance reading was obtained at 450nm every minute for 10 min at room temperature. SOD activity of the samples were plotted against SOD standard curve.

### *In silico* prediction of protein-protein interaction

Molecular Docking: Crystal structures of gelsolin (PDB code: 3FFN, Chain A) and Cu/ZnSOD (PDB code: 1PU0, Chain A) were obtained form Protein Data Bank. Molecular docking between Gelsolin and Cu/ZnSOD was performed using PatchDock with default cut-off values [73]. PatchDock is a geometry-based molecular docking algorithm, which aims at finding docking confirmations based on the molecular shape complementarity.

Molecular dynamics: Molecular dynamics simulations were performed using the GROMACS version 4.5.3 with OPLS force field [74]. The heterodimer model (Gelsolin-Cu/ZnSOD complex) was placed in a cubic box with the box-edges at least 10nm apart from the protein surface. The system was solvated with Simple Point Charge (SPC216) water molecules and appropriate number of counterions was added in the box to neutralize the system. In order to remove the possible clashes between atoms, the energy minimization was set to run for 50000 steps or until convergence to machine precision. After energy minimization, simulations were performed for 100 ps at constant temperature and pressure with periodic boundary conditions, particle-mesh Ewald summation, and a 2-fs time step to heat and equilibrate the system. Then the system was subjected to production of MD simulations for 10 ns. Structures were saved every 2 ps for analysis. The output files from the GROMACS 4.5.3 was analyzed using XMGRACE software. The overall stability of the dimer was measured by estimating the root mean square deviation (RMSD) of the molecule and its radius of gyration (Rg).

Interactions (hydrogen bonds and hydrophobic forces) were analyzed at the interface in each simulated structure. Interactions between hydrophobic side chains are identified using a distance cut off of 5 Angstrom between apolar groups in the apolar side chains [75]. The hydrogen bonds formed between subunits are identified using HBOND program which is a part of JOY suite [76]. The interactions that exist in at least 60% of the simulated structures were considered as dynamically stable and used for the interpretation of stability.

### Data preprocessing of microarray gene expression

Gene expression microarray data of colon cancer on U133A or U133Plus2 platforms were downloaded from Gene Omnibus (GEO), including GSE10961 (n = 18), GSE12945 (n = 62), GSE13067 (n = 74), GSE13294 (n = 155), GSE14333 (n = 290), GSE15960 (n = 12), GSE17536 (n = 177), GSE17537 (n = 55), GSE18088 (n = 53), GSE18105 (n = 77), GSE20916 (n = 101), GSE23878 (n = 35), GSE24514 (n = 34), GSE26682 (n = 331), GSE31595 (n = 37), GSE33113 (n = 90), GSE4045 (n = 37), GSE5851 (n = 80), GSE8671 (n = 32), and GSE9348 (n = 70). Robust Multichip Average (RMA) normalization was performed on each dataset. The normalized data was compiled and subsequently standardized using ComBat to remove batch effect [77]. The standardized data yielded a dataset of 1,820 colon carcinoma [78]. A non-parametric test (Spearman's rank correlation) was used to access the correlation and significance of GSN (gelsolin) with other factors.

### Sandwiched Enzyme-linked immunosorbent assay (ELISA)

Cells were cultured in serum free media with or without 5μM DPI for 8 h. Conditioned media was harvested for ELISA. ELISA was performed following standard sandwiched ELISA methods using the uPA DuoSet ELISA kit (R&D Systems, MN, USA) according to the maufacturer's protocol. ELISA plates were coated with 100μL capture antibody overnight. Wells were then washed thoroughly (3-5 times) with 400μL of wash buffer, followed by blocking the plates with 300μL reagent diluent for 1 h. Wells were washed three times with 1X wash buffer (0.05% PBST). 100μL of samples were dispensed to the wells and incubated for 2 h at room temperature. Wells were washed and 100μL of Streptavidin-HRP was added to each well and incubated for 20 min at room temperature. This was followed by adding 100μL of substrate solution and again incubated for 20 min at room temperature. The reaction was stopped by adding 50μL of 1M H_2_SO_4_. The optical density of each well was measured using ASYS UVM 340 microplate reader at 450nm, using 570nm as reference wavelength.

### Microscopy

Cell counting and images for Invasion assay were obtained using Carl Zeiss Axiovert 40 CFL - Inverted Microscope and images were processed Image J. Visualization and images for the *In situ* Proximity Ligation Assay were processed using Olympus DP72 microscope and cellSens software.

### Intracellular calcium measurement

Vector control cells and gelsolin-overexpressing cells (C1) were were incubated with 5μM Fura2-AM for 30 minutes in calcium buffer (1.8% glucose, 2% BSA, 1mM CaCl_2_, 145mM NaCl, 5mM KCl, 1mM MgCl_2_, 10mM Hepes pH7.4). Cells were then washed twice with calcium buffer and subjected to ratiometric analysis of Fura2-AM fluorescence using Xcellence software under Olympus IX73 fluorescence microscope. Intracellular calcium level was measured by fluorescence ratio of 340nm/380nm (excitation at 340nm and 380nm and emission at 540nm) at 0.8s interval. Average value of 340nm/380nm ratio over 3 minutes was calculated, and the fold changes of ratio compared to vector control cells were calculated.

### Statistical analysis

All statistical analyses were performed using a two tailed Student's *t*-test. Differences between sample means were considered statistically significant when p-value < 0.05.

## SUPPLEMENTARY MATERIAL FIGURES


