# Supplementary material for: Gelsolin-Cu/ZnSOD interaction alters intracellular reactive oxygen species levels to promote cancer cell invasion
Source: Oncotarget. 2016 Jul 6;7(33):52832–48. doi: 10.18632/oncotarget.10451 (PMC5288152; doi:10.18632/oncotarget.10451)
Supplement: Supplementary file 1 [file oncotarget-07-52832-s001.pdf]

Gelsolin-Cu/ZnSOD interaction alters intracellular reactive oxygen species levels to promote cancer cell invasion

Supplementary Material

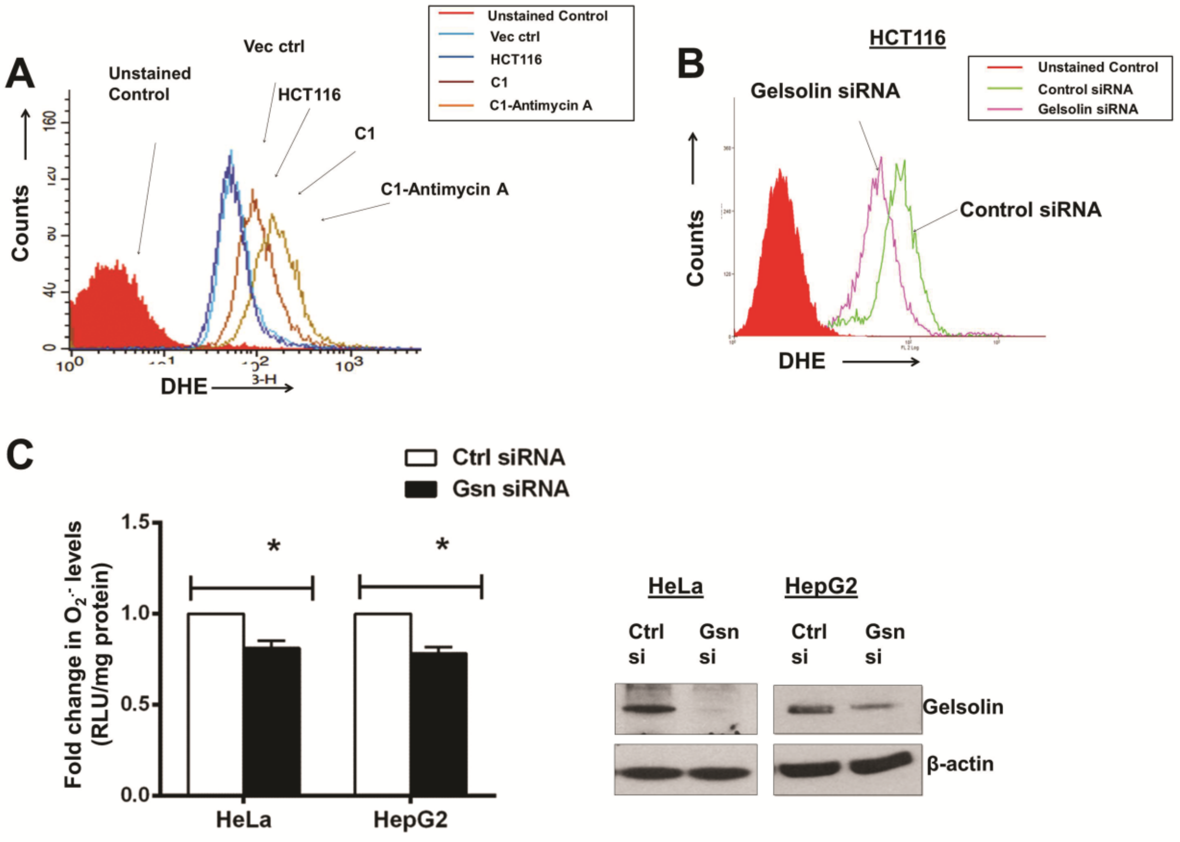

Supplementary Figure 1: Superoxide ($O_2^{.-}$) levels were detected following gelsolin modulation using the fluorescent Dihydroethidium (DHE) staining and the chemiluminescent-based Lucigenin assays. (A) DHE staining shows that gelsolin-overexpressing C1 and C8 cells have higher $O_2^{.-}$ levels compared with empty vector control cells and wild-type HCT116 indicated by right shift in the histogram above. Treatment of cells with Antimycin A serves as a positive control for the detection of $O_2^{.-}$. (B) $O_2^{.-}$ levels were compared in HCT116 cells between gelsolin-knockdown and the control siRNA-treated cells using DHE staining. Knockdown of gelsolin in HCT116 lowers $O_2^{.-}$ levels as indicated by pink histogram that shifts to the left. (C) Left panel: Lucigenin assay was used to assess $O_2^{.-}$ levels in HeLa and HepG2 cells. siRNA-mediated knockdown of gelsolin in HeLa and HepG2 cells results in decreased levels of $O_2^{.-}$ when compared to the control siRNA. Right panel: Western blot showing gelsolin-knockdown in HeLa and HepG2 cells. Flow cytometry data shown is a representative from three independent experiments. *p-value <0.05 versus controls using a two tailed Student's

t-test. Values (mean ± SD) are expressed as fold over the empty vector control or the control siRNA, which was arbitrarily set as one. The western blot gel picture is a representative image from three independent experiments.

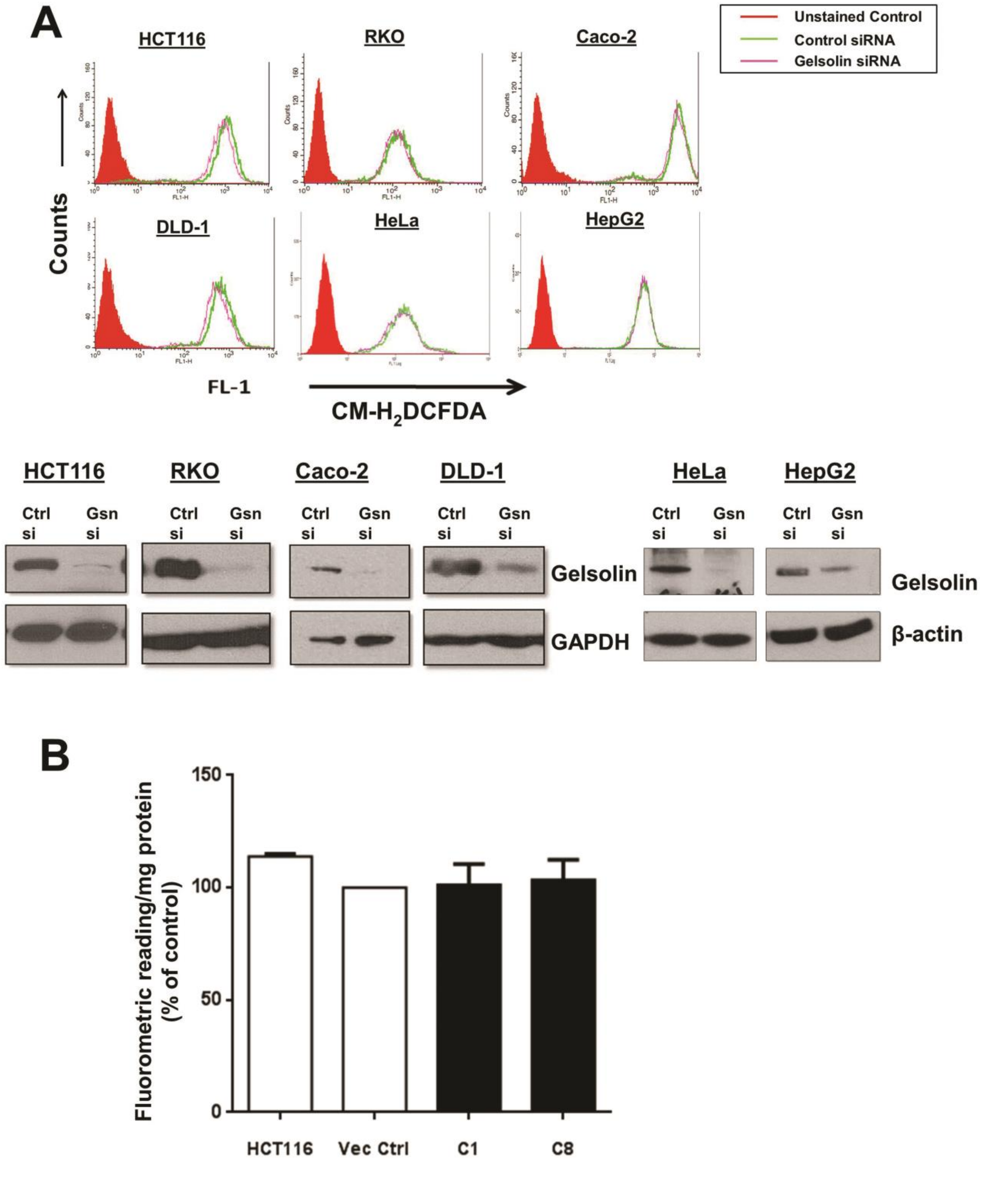
A
HCT116
RKO
Caco-2
DLD-1
HeLa
HepG2
Unstained Control
Control siRNA
Gelsolin siRNA
Counts
FL-1
CM-H2DCFDA
Ctrl si
Gsn si
Gelsolin
GAPDH
β-actin
B
Fluorometric reading/mg protein (% of control)
150
100
50
0
HCT116
Vec Ctrl
C1
C8

Supplementary Figure 2 (A-B): Gelsolin-knockdown does not affect the levels of other ROS (H_2O_2,$^{\cdot}$OH and HOCl). (A) Upper panel: Gelsolin was knocked down in HCT116, RKO, Caco-2, DLD-1, HeLa and HepG2 cancer cell lines and ROS levels were compared between gelsolin-knockdown cells and control siRNA-treated cells using CM-H_2DCFDA. Histogram shows the intensity of CM-H_2DCFDA staining measured at FL-1 channel. Lower panel: Western blots showing the efficiency of gelsolin-knockdown in HCT116, RKO, Caco-2, DLD-1, HeLa and HepG2 cells. GAPDH and Beta-actin were used as an internal loading control. (B) Overexpression of gelsolin expression does not alter H_2O_2 levels. Amplex Red was used to assess H_2O_2 levels in gelsolin-overexpressing cells. Bar charts showing relative fluorometric reading of Amplex Red dye. Values (mean ± SD) are expressed as percentage over the empty vector control. The western blot gel pictures are representative images from three independent experiments.

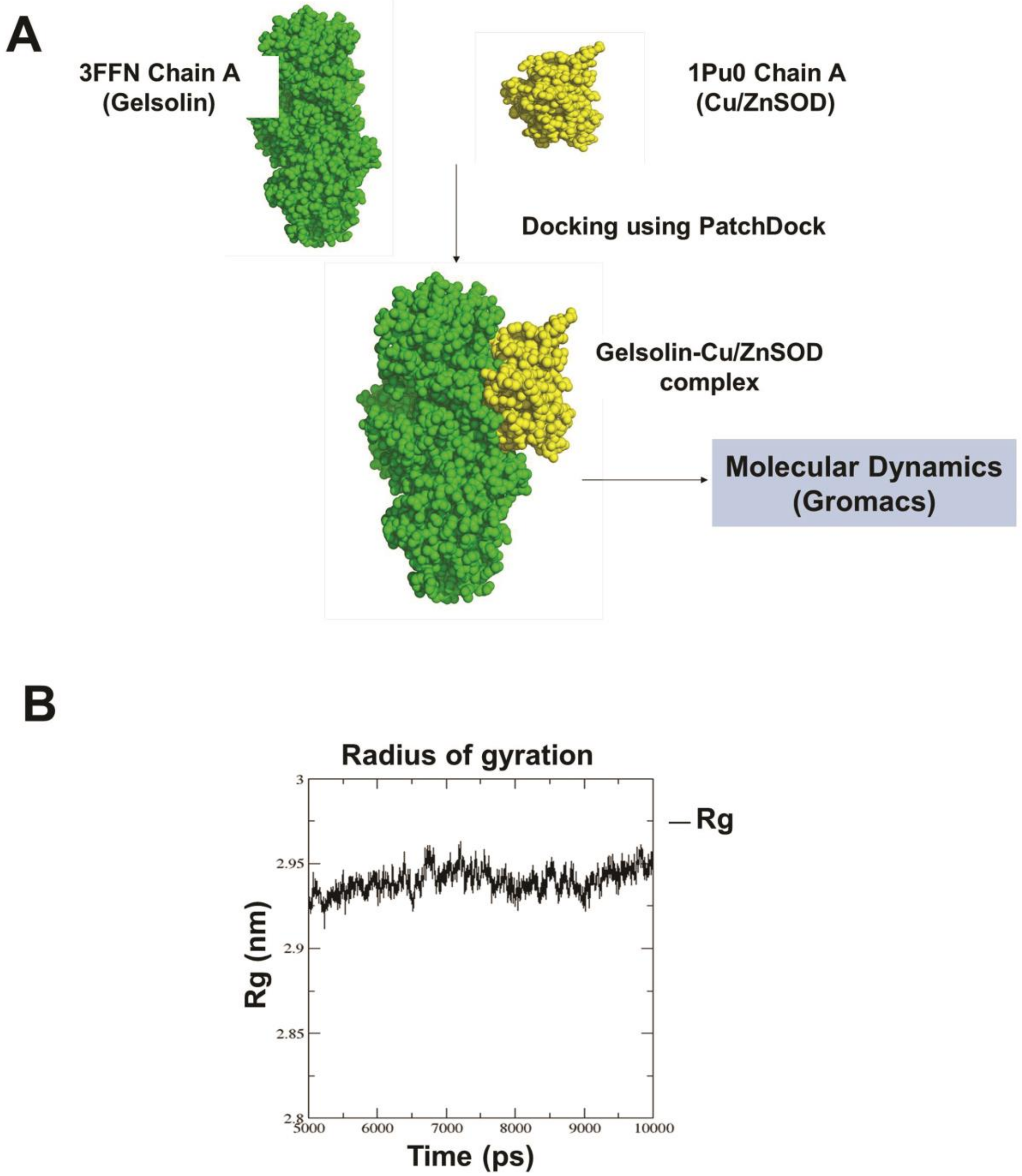

A
3FFN Chain A
(Gelsolin)
1Pu0 Chain A
(Cu/ZnSOD)
Docking using PatchDock
Gelsolin-Cu/ZnSOD
complex
Molecular Dynamics
(Gromacs)
B
Radius of gyration
— Rg
Rg (nm)
3
2.95
2.9
2.85
2.8
5000
6000
7000
8000
9000
10000
Time (ps)

Supplementary Figure 3: Docking and moledular dynamics simulation of 3-dimenstional structures of Gelsolin-Cu/ZnSOD. (A) 3-dimensional structure of Gelsolin-Cu/ZnSOD complex obtained using PatchDock. The green structure corresponds to gelsolin (PDB: 3FFN, chain A) and the yellow structure represents the Cu/ZnSOD structure (PDB: 1PU0 Chain A). (B) Molecular dynamics was performed for gelsolin-Cu/ZnSOD complex that mimics physiological conditions. Fluctuation in the radius of gyration (Rg) around a stable value is taken as a measure of protein stability. If a protein is stably folded, it will likely maintain a relatively steady value of Rg. If a protein unfolds, its Rg will change over time. The above curve shows a steady value of Rg for upto 10 ns indicating the compactness of the gelsolin-Cu/ZnSOD complex structure.

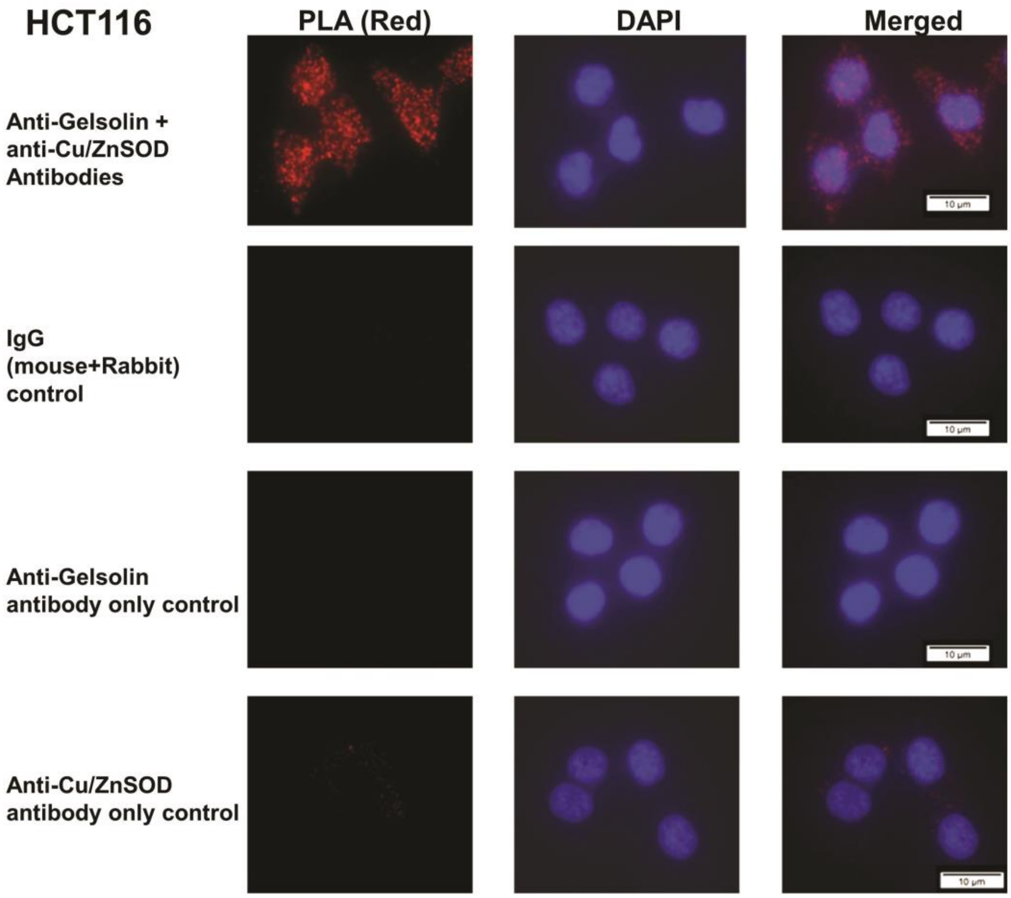

Supplementary Figure 4: Gelsolin and Cu/ZnSOD lie in close proximity to each other. Proximity ligation assay (PLA) was performed in HCT116 cells. PLA signals in red fluorescence were detected when cells were treated with both gelsolin and Cu/ZnSOD antibodies. PLA signals were not detected in isotype control cells as well as in single antibody-treated cells. Images were captured using Olympus DP72 microscope and cellSens software at 60X. Scale bar represent 10µm. Data is a representative of three independent experiments.

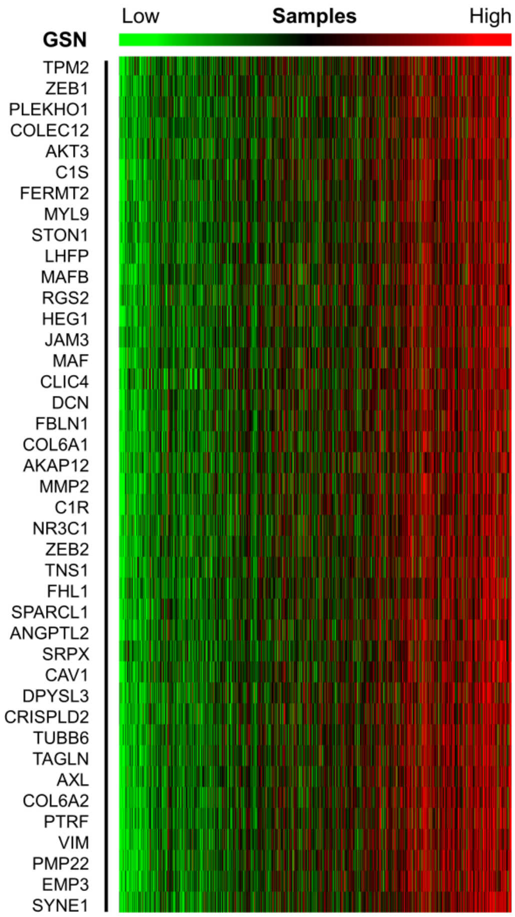

Supplementary Figure 5: Correlation of gelsolin gene expression with EMT genes. Correlation of gelsolin expression with EMT signature genes across 1820 colorectal cancer samples. Heatmap is used to show the correlation of those EMT signature genes [78] significantly correlated with gelsolin in the terms of gene expression. Colorectal cancer samples were aligned from the lowest (green) to highest (red) gelsolin gene expression.

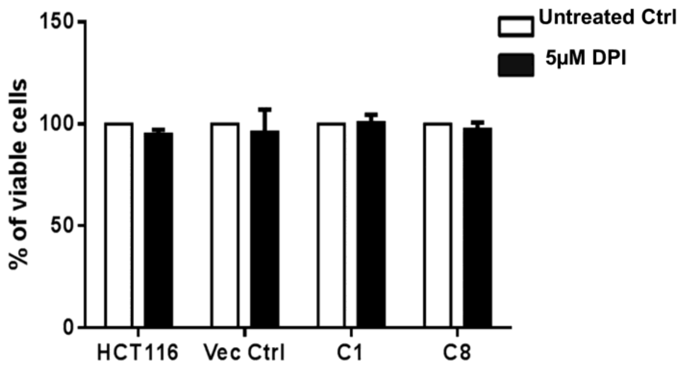

Supplementary Figure 6: DPI has minimal effect on HCT116 cell viability in cell invasion. To confirm the effect of DPI on gelsolin-induced cell invasion, gelsolin-overexpressing cells were treated with 5µM DPI for 24 h and cell viability was assessed using the Trypan Blue Exclusion cell counting method. Approximately $4x10^5$ cells were seeded in a 12 well plate with or without 5µM DPI in media containing 1% FBS for 24 h. Cells were detached from the wells and appropriate dilutions were made for cell counting. The diluted cell suspension was then mixed in equal ratio with trypan blue dye. 10µL of this mixture was loaded onto a haemocytometer and the number of viable cells that did not take up the dye were counted. No significant difference in cell viability was observed between control group and DPI treated cells. Values (mean ± SD) are expressed as percentage over the empty vector control.

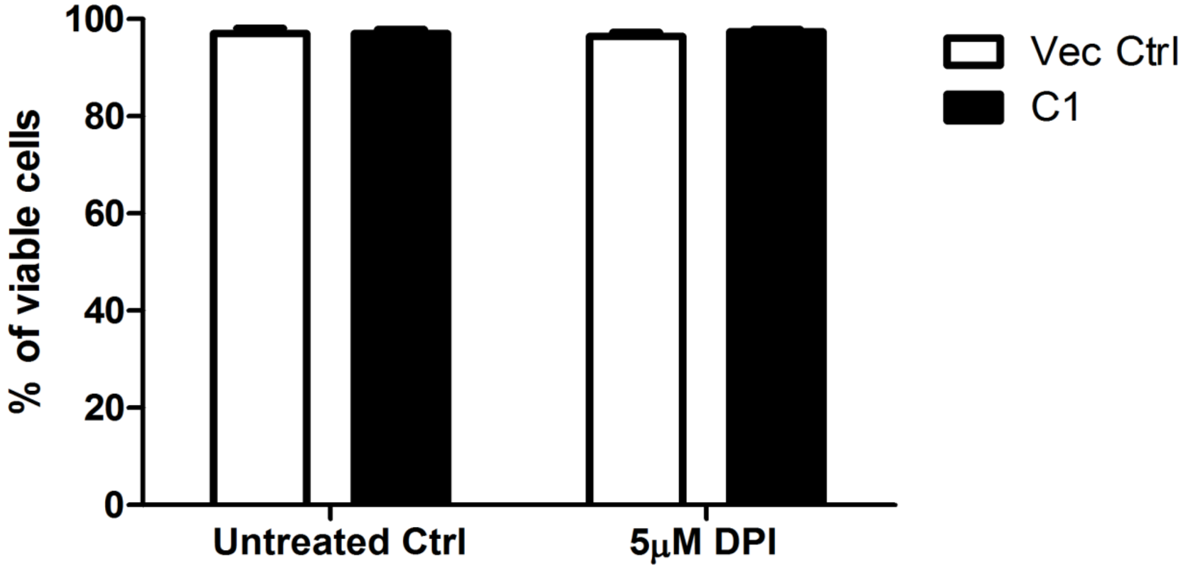

Supplementary Figure 7: DPI has minimal effect on HCT116 cell viability in uPA secretion. To confirm the effect of DPI on gelsolin-induced uPA secretion, cells were seeded in 12-well plates and treated with 5μM DPI in serum-free medium for 8h, and cell viability was assessed using the Trypan Blue Exclusion cell counting method. Cell viability was calculated as percentage of viable cells that did not take up the dye. No significant difference in cell viability was observed between control group and DPI treated cells. Values (mean ± SEM) are expressed as percentage over the empty vector control.

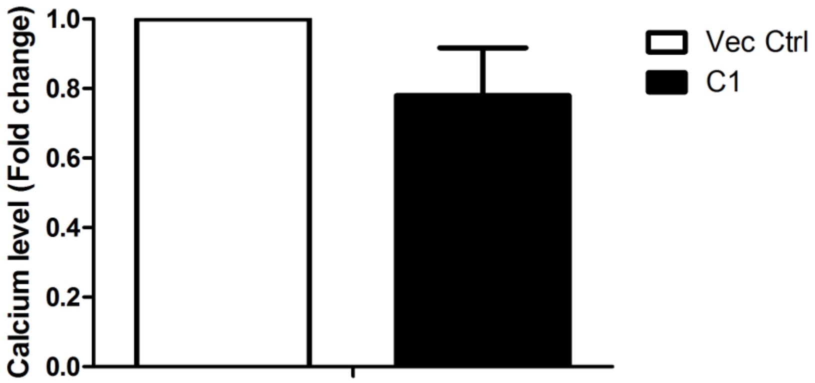

Supplementary Figure 8: Calcium levels in gelsolin-overexpressing and vector control HCT116 cells. Intracellular calcium levels of vector control cells and gelsolin-overexpressing cells (C1) were measured by Fura2-AM dye fluorescence. Cells were incubated with 5µM Fura2-AM for 30 minutes in calcium buffer before being subjected to ratiometric analysis of Fura2-AM fluorescence using Xcellence software under Olympus IX73 fluorescence microscope. Intracellular calcium level was measured by fluorescence ratio of 340nm/380nm (excitation at 340nm and 380nm and emission at 540nm) at 0.8s interval. Average value of 340nm/380nm ratio over 3 minutes was calculated, and the fold changes of ratio compared to vector control cells were presented (Mean±SEM of at least three independent experiments).
